# A Spontaneous Inversion of the X Chromosome Heterochromatin Provides a Tool for Studying the Structure and Activity of the Nucleolus in *Drosophila melanogaster*

**DOI:** 10.3390/cells11233872

**Published:** 2022-12-01

**Authors:** Tatyana D. Kolesnikova, Mikhail S. Klenov, Alina R. Nokhova, Sergey A. Lavrov, Galina V. Pokholkova, Veit Schubert, Svetlana V. Maltseva, Kevin R. Cook, Michael J. Dixon, Igor F. Zhimulev

**Affiliations:** 1 Institute of Molecular and Cellular Biology SB RAS, 630090 Novosibirsk, Russia; 2 Faculty of Natural Sciences, Novosibirsk State University, 630090 Novosibirsk, Russia; 3 Department of Molecular Genetics of the Cell, Institute of Molecular Genetics of National Research Centre “Kurchatov Institute”, 123182 Moscow, Russia; 4 Leibniz Institute of Plant Genetics and Crop Plant Research Gatersleben, 06466 Seeland, Germany; 5 Bloomington Drosophila Stock Center, Department of Biology, Indiana University, Bloomington, IN 47405, USA

**Keywords:** heterochromatin, nucleolus, inversion, *Rif1*, *Drosophila melanogaster*, polytene chromosomes, underreplication, *In(1)sc^8^*

## Abstract

The pericentromeric heterochromatin is largely composed of repetitive sequences, making it difficult to analyze with standard molecular biological methods. At the same time, it carries many functional elements with poorly understood mechanisms of action. The search for new experimental models for the analysis of heterochromatin is an urgent task. In this work, we used the *Rif1* mutation, which suppresses the underreplication of all types of repeated sequences, to analyze heterochromatin regions in polytene chromosomes of *Drosophila melanogaster*. In the *Rif1* background, we discovered and described in detail a new inversion, *In(1)19EHet*, which arose on a chromosome already carrying the *In(1)sc^8^* inversion and transferred a large part of X chromosome heterochromatin, including the nucleolar organizer to a new euchromatic environment. Using nanopore sequencing and FISH, we have identified the eu- and heterochromatin breakpoints of *In(1)19EHet*. The combination of the new inversion and the *Rif1* mutation provides a promising tool for studies of X chromosome heterochromatin structure, nucleolar organization, and the nucleolar dominance phenomenon. In particular, we found that, with the complete polytenization of rDNA repeats, the nucleolus consists of a cloud-like structure corresponding to the classical nucleolus of polytene chromosomes, as well as an unusual intrachromosomal structure containing alternating transcriptionally active and inactive regions.

## 1. Introduction

A significant part of eukaryotic genomes consists of constitutive heterochromatin, which is mainly located adjacent to the centromeres. Although pericentromeric heterochromatin regions are largely composed of repetitive sequences, they carry vital loci, such as ribosomal DNA (rDNA) repeats, as well as many functional elements with poorly understood mechanisms of action.

Heterochromatin accounts for ~59 megabases (Mb) of the 176-Mb genome of the *D. melanogaster* female, and the 41-Mb male Y chromosome is entirely heterochromatic [1,2]. In the heterochromatin of the major autosomes (chromosomes 2 and 3), satellite DNA alternates with islands of complex DNA containing many vital genes. The genetic organization of the *D. melanogaster* X chromosome appears to differ markedly from that of autosomes. The only vital locus mapped within the X chromosome heterochromatin (Xh) is a repetitive rDNA cluster encoding 18S, 5.8S and 28S rRNAs [3,4]. While boundaries between eu- and heterochromatin can be clearly delineated in autosomes, it is quite difficult to identify the eu-/heterochromatin boundary of the X chromosome since a gradient of heterochromatin properties is observed (reviewed in [5]). In addition, an extended block of constitutive heterochromatin in section 20BC of the X chromosome is followed by a gene-rich section, which is attributed by different authors either to eu- or heterochromatin. Although most and probably all of the Xh located distal to the rDNA locus is not required for viability or fertility, this region is thought to be biologically significant. In particular, it influences the expression of the maternal-effect mutation *abnormal oocyte* (*abo*) located within the chromosome 2 euchromatin [6] and carries a group of still poorly characterized genetic loci that are thought to be composed of repetitive elements: *compensatory response (cr)*, *collochore (col)*, *and Ribosomal exchange (Rex)* with its suppressor (reviewed in [7]).

rDNA clusters are located both in Xh and on the Y chromosome, being about 2.2 and 2.8 Mb long, respectively, although their sizes vary greatly among *D. melanogaster* lines. The basic rDNA units encoding 18S, 5.8S, 2S and 28S rRNAs are approximately 12 kb long but can differ in intergenic spacer (IGS) length, as well as in the presence of insertions of R1 and R2 retrotransposons [8,9,10,11]. The rDNA copy number ranges from 80 to 600 [10,12,13,14], whereas individuals with ~130 rDNA copies or fewer display a *bobbed* mutant phenotype, which is represented by shortened and abnormally thin scutellar bristles and by delayed development [15]. rDNA clusters are dynamic genetic elements in which the rDNA copy number can change over generations or cell divisions, subject to the control of a recovery mechanism in the germline [16]. An actively transcribed rDNA cluster forms the nucleolus, a membrane-less organelle that, in addition to the biogenesis and assembly of ribosomes, performs many other functions [17,18]. In various organisms, the phenomenon of inactivation of individual rDNA clusters, known as “nucleolar dominance”, has been described. In males of *D. melanogaster*, the Y chromosome rDNA locus is usually thought to dominate over the X chromosome locus [19,20,21]. The choice of an active rDNA cluster is a complex phenomenon, which depends, among other things, on *cis*-acting factors, such as the distal pericentromeric heterochromatin of the X chromosome [21,22].

Inversions with breakpoints in heterochromatin serve as important tools for the exploration and mapping of functional elements in Xh [4,23,24]. The first classical inversions, including *In(1)sc^8^*, were obtained by irradiation [25,26,27]. Later, researchers obtained more complex rearrangements from recombination between the inverted chromosomes, including minichromosomes, deletions, multiple inversions, and balancer chromosomes. Many X chromosome balancers include an *In(1)sc^8^* inversion [28], which inverts almost the entire X chromosome, with one breakpoint near the telomere in section 1B2 (*X:382,384–382,384* (*In(1)sc^8^*:bk1)) and a second in the h32 block near the centromere (*X:21,368,973–23,542,271* (*In(1)sc^8^*:bk2)). The heterochromatin breakpoint is located within repeats of 1.688 satellite DNA, which cover about half of the X chromosome pericentromeric heterochromatin [28]. X chromosome eu/heterochromatin inversions are also the main classical tools for studying position-effect variegation [29,30,31,32,33], but few have been characterized molecularly [28,34]. Third-generation sequencing platforms, in particular, Oxford Nanopore Technologies, allow us to obtain sequence reads up to hundreds of kb, which are suitable for detailed analysis of inversion breakpoints, particularly those flanked by repeated DNA [35,36]. Using this approach, Solodovnikov and Lavrov [36] recently characterized the breakpoints of the *In(1)w^m4^* rearrangement, which has been used more than any other inversion in studies of position-effect variegation.

Since heterochromatin has a repeated nature, cytological methods remain relevant and important for its study. In *Drosophila* polytene chromosomes, heterochromatin is subject to underreplication that can be suppressed by several mutations. The strongest effect was found in *Rif1* gene mutants, in which all types of repeated heterochromatin sequences, including satellite DNA, were polytenized [37,38]. In the *Rif1* mutants, as in the wild type, most of the heterochromatin becomes replicated during the late S phase, but a significantly increased number of heterochromatin replicons was noted. Therefore, it was assumed that *Rif1* regulates the activation probability of heterochromatic origins in the satellite DNA region [38]. Complete polytenization of Xh allowed us, for the first time, to visualize the 11 Mb 1.688 g/cm^3^ (hereinafter, 1.688) satellite block and AATAT and AAGAG satellites adjacent to the centromere. Different satellite sequences appeared to vary significantly in DAPI staining intensity. The alternation of DAPI-positive and -negative zones allowed researchers to isolate many discrete blocks in heterochromatin in *Rif1* polytene chromosomes [38]. In situ hybridization indicated a correspondence between these blocks and DAPI-positive satellites (AATAT, 1.688) or DAPI-negative satellites (AAGAG and others).

The polytene chromosomes of *Rif1* mutants can also be useful for visualizing Xh adjacent to the nucleolus. In *Rif1* mutants, the nucleolus-forming region does not appear to be separated from the chromosome by underreplicated chromatin in squash preparations. Therefore, a typical structure characteristic of the nucleolus, a cloud of diffuse chromatin that stains for rDNA and has characteristic contours on phase-contrast images, appears attached to a specific region of the chromosome. It remains unclear whether this zone of the chromosome is part of the nucleolar organizer and whether it is transcriptionally active [38].

In this work, we have discovered and analyzed in detail a new inversion *In(1)19Ehet* that arose on an X chromosome carrying the classical *In(1)sc^8^* inversion, which moves a large portion of the Xh from its normal position adjacent to the centromere to a position near the distal X tip. The new double-inversion transferred most of this heterochromatin region much deeper into euchromatin. As a result, a large block of Xh, including the nucleolar organizer, is located separately from the massive heterochromatic chromocenter on polytene chromosome preparations. When placed in a *Rif1* mutant background, this double-inversion chromosome provides an exceptional opportunity to analyze Xh structure, nucleolus activity and the effects of repositioning heterochromatin within the nucleus.

## 2. Materials and Methods

### 2.1. Flies

The fly stocks mentioned in this work are described in Table 1. For all types of cytological analysis of polytene chromosomes, larvae were reared on enriched semolina (36 g/L) medium with raisins at 18 °C. For other experiments, flies were reared on a standard medium at 25 °C.

### 2.2. Morphological Analyses and Microscopy of Polytene Chromosomes

Polytene chromosomes were fixed and stained with aceto-orcein by the method of Zhimulev et al. [39]. Phase contrast images were acquired with an Olympus BX51 microscope using a 100×/1.30 Uplan FI objective and a DP52 camera (Olympus, Tokyo, Japan). The same fixation procedure was used to prepare polytene chromosomes for three-dimensional, structured illumination microscopy (3D-SIM), which was performed with a Zeiss Elyra PS.1 microscope system using a Plan-Apochromat 63×/1.4 oil objective and the ZENBlack 2.1 software (Carl Zeiss GmbH, Jena, Germany). Image stacks for each fluorochrome were generated with 561, 488 and 405 nm laser excitation and appropriate emission filters [40].

### 2.3. MinIon Sequencing

High molecular weight DNA (HMW DNA) was extracted from ~100 larval imaginal discs and brains or salivary glands using a variation on the standard proteinase K treatment—phenol-chloroform extraction—ethanol precipitation protocol [36]. Liquid handling during DNA isolation was carried out gently (without vortexing steps and using cut-off pipette tips) to avoid shearing the DNA. The concentration of prepared DNA was measured using a Qubit fluorimeter (dsDNA broad range kit, Thermo Fisher Scientific, Waltham, MA, USA) [41]. The sequencing library was prepared from 0.5 μg of DNA using a Ligation Sequencing Kit (SQK-LSK109) and Native Barcoding Expansion (EXP-NBD104) from Oxford Nanopore Technologies (ONT), Oxford, UK, according to the manufacturer’s protocol. A MinIon R 9.4.1. flowcell (ONT) was used to sequence the library without basecalling. The fast5 files generated by sequencing were basecalled using the dna_r9.4.1_450bps_hac profile and barcode demultiplexing in Guppy (version 3.5.2) running on a standalone GPU-enabled server.

### 2.4. Nanopore Sequencing Data Treatment and Breakpoint Detection

Reads with Q < 7 were filtered out in the course of basecalling. The resulting FASTQ files were loaded to the local Galaxy instance for quality checks and analysis. The default settings in Porechop (https://github.com/rrwick/Porechop accessed on 12 May 2022) were used to trim adapters. Reads with a middle adapter were split. Four samples of DNA from the *In(1)sc^8^, In(1)19EHet* flies were sequenced. These datasets were combined into one, and the reads of less than 15 kb in size were filtered out using NanoFilt (Galaxy Version 0.1.0). The resulting dataset contains ~2.9 Gb of nucleotides with N50 = 39722. Reads from the processed dataset were aligned to the *Drosophila melanogaster* reference genome (release 6.22) using the Oxford Nanopore read-to-reference profile (minimap2-x map-ont) in Minimap2 [42], and the bam output file was displayed for analysis in a local copy of the UCSC Genome Browser (https://genome.ucsc.edu/ accessed on 12 May 2022). FASTQ output files were converted to FASTA format using a Galaxy FASTQ-to-FASTA script [43].

In order to find the original breakpoints of *In(1)sc^8^* [28], we visually inspected the *chrX:382,384–382,389* region in the UCSC Genome Browser and found a gap in coverage in the predicted position. To extract the reads overlapping the breakpoint and containing heterochromatin beyond the breakpoint, we picked two short fragments of DNA leftmost and rightmost to the breakpoint (sc8_bp_left_left, *chrX:381,979-382,378* and sc8_bp_left_right, *chrX:382,391-382,719*) and blasted the read dataset against these sequences. The BLAST results were then filtered based on the read length and the distance between the end of the read and the query match position. The two longest reads, encompassing ~30 kb of heterochromatin sequences (bda1abf7-651b-43e0-b37a-5f8076b4eb18 and 043b5081-ff6e-4b31-9806-8ef1052143dc), were selected for mapping via RepeatMasker (http://www.repeatmasker.org/cgi-bin/WEBRepeatMasker accessed on 12 May 2022). The output was converted to bed format manually and displayed in IGV (https://software.broadinstitute.org/software/igv/ accessed on 12 May 2022) with color coding for different repeat types. Additionally, several shorter reads from this region were examined in RepeatMasker in order to check the reliability of the received feature map, and the distribution of heterochromatin elements was confirmed. The position of *In(1)19EHet* rearrangement was inspected similarly to *In(1)sc^8^*. The longest reads overlapping *In(1)19EHet* breakpoints are 6ea4eb01-9107-4c78-9973-da248451fe66 and 53be4a6f-1768-418d-bdd9-33de00fca102.

### 2.5. PCR Primers and FISH Probes

To verify the results of inversion mapping obtained using nanopore sequencing, PCR reactions were performed with various pairwise combinations of the following primers:

- P1 5′-ACTTTGATGCCTGCTCCAGT-3′;
- P2 5′-ATTGACACAACCCATTTAAGAG-3′;
- P3 5′-GGGCAGGTTCGAGGTTGGGAAGC-3′;
- P4 5′-CCTTTGCCAGTTGAGTTTTCTATGCCG-3′;
- P5 5′-GCCATTGTCCAGCAATCGCCAAA-3′;
- P6 5′-GCCAAGTACTTTGCCATCTTTCG-3′;
- P7 5′-GTCTGGAGCGAGAGCGGCCCTC-3′;
- P8 5′-CGCGCACGCTTTCTGCAAAA-3′;
- P9 5′-CGCTTAAGAGCGTAAAATGCATGGAG-3′.

The 19E FISH probe, corresponding to a 1151 bp genome fragment crossing the *In(1)19EHet* inversion breakpoint, was generated by PCR with the primers P1 and P2.

The 19EL FISH probe, corresponding to an 858 bp genome fragment to the left of the *In(1)19EHet* breakpoint, was generated by PCR with the primers 5′-TGGTGTCGGTTATCAGTTACG-3′ and 5′-TAGCACAATGTCAACAGTTGC-3′.

The 19ER FISH probe, corresponding to a 1091 bp genome fragment to the right of the *In(1)19EHet* breakpoint, was generated by PCR with the primers 5′-TGATGGTGAGCACTCATTTAG-3′ and 5′-CGGGCATTAGACTATGAATCA-3′.

The probe for heterochromatin-specific variants of the 359-bp satellite *1.688Xhet* [44] was amplified with the primers 5′-TAGGGATCGTTAGCACTGGT-3′ and 5′-ACGAGCTCAGTGAGATATGA-3′.

For rDNA detection, a DNA clone with a 0.9-kb HindIII fragment of the *28S ribosomal RNA* (*rRNA*) gene [45] was used.

DNA clones were random-prime labeled with a Klenow fragment using Tamra-5-dUTP or FLu-12-dUTP (Biosan, Novosibirsk, Russia).

### 2.6. Fluorescence In Situ Hybridization (FISH) and Indirect Immunostaining

Polytene chromosome preparation, FISH analysis and indirect immunostaining were performed according to the protocol described in [38].

Rabbit polyclonal anti-Fibrillarin (Abcam, ab5821, 1:5000) or guinea pig anti-Udd (1:300, [46], kindly provided by M. Buszczak) primary antibodies and Alexa Fluor 488 anti-guinea pig or/and Alexa Fluor 568-conjugated goat anti-rabbit secondary antibodies (1:500; Thermo Fisher Scientific, Waltham, MA, USA) were used for immunostaining.

### 2.7. EU Incorporation and Detection

Salivary glands were dissected and stored in 1 × PBS (137 mM NaCl, 3 mM KCl, 8 mM NaH_2_PO_4_, 2 mM KH_2_PO_4_). EU incorporation was carried out in a 0.2 mM EU (Sigma Aldrich, Burlington, MA, USA) solution in 1 × PBS for 20 min. Further procedures for preparing polytene chromosome squashes and EU detection were performed according to the EdU detection protocol described in [38].

### 2.8. Data Availability

The datasets with the results of Nanopore sequencing of *In(1)sc^8^, In(1)19EHet* fly DNA are available from the SRA (SRR22071703, SRR22071702, SRR22071691, SRR22071690).

## 3. Results

### 3.1. Detection of a New Inversion That Spontaneously Appeared in BDSC Line #798 with In(1)sc^8^ Inversion

**Figure 1:**
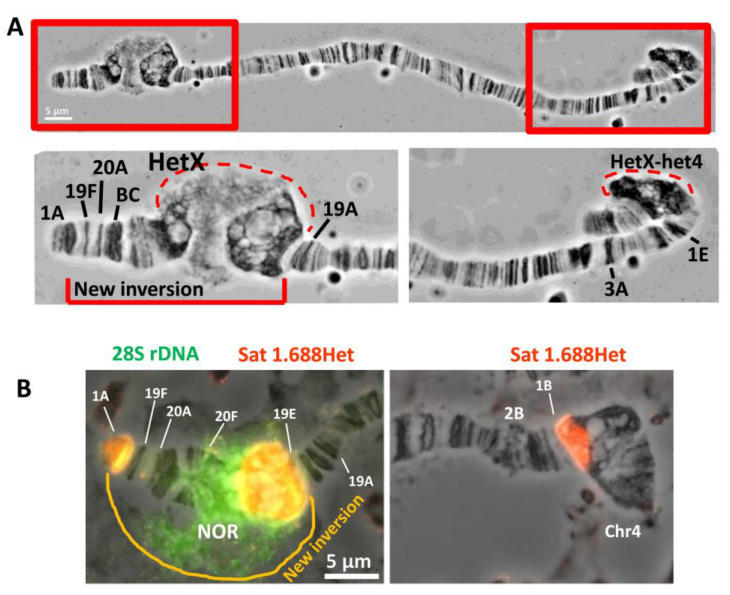
Detection and cytological mapping of the new X-chromosomal eu/heterochromatin inversion. (**A**) Polytene X chromosome of the new line carrying the *Rif1^1^* mutation, the *In(1)sc^8^* inversion, and the additional inversion named *In(1)19EHet* (*In(1)sc^8^ + 19EHet; Rif1^1^*). The enlarged end segments of the chromosome highlighted in red rectangles are shown at the bottom. Of note, in *Rif1^1^* mutants, proximal X heterochromatin is always combined into a single block with chromosome 4 heterochromatin [38]. Aceto-orcein staining, phase contrast. (**B**) DNA FISH of the *In(1)sc^8^ + 19EHet*; *Rif1^1^* polytene X chromosome with probes to satellite 1.688Het (red) and 28S rDNA (green).

**Figure 2:**
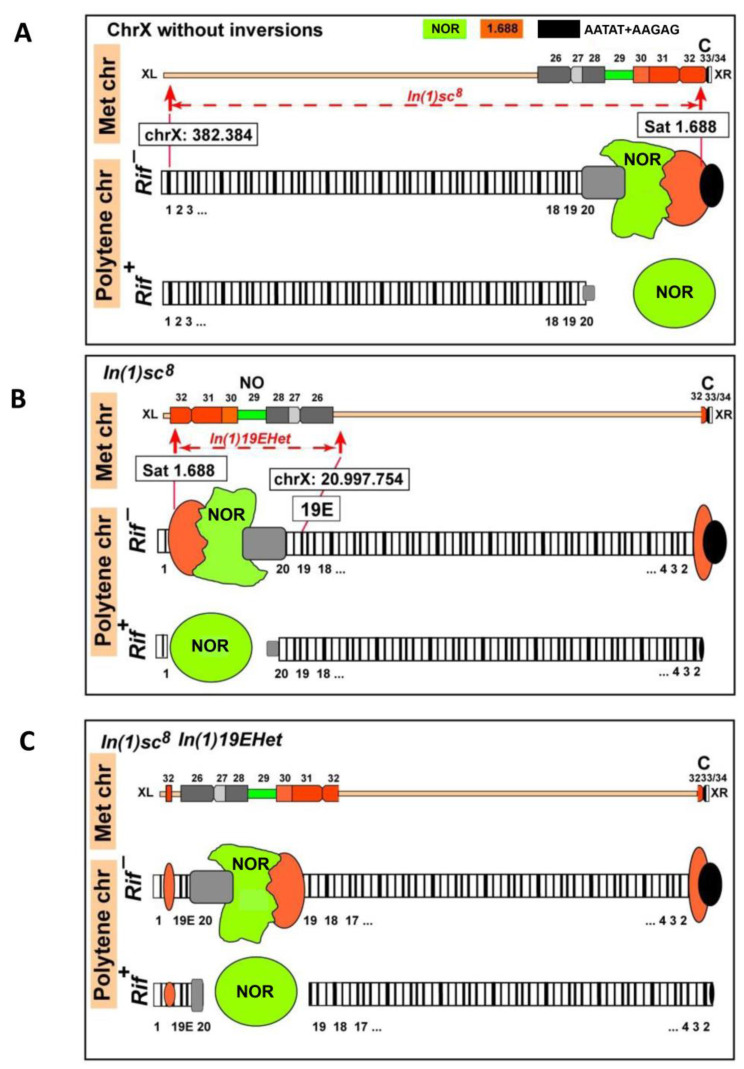
Schematic representation of metaphase and polytene X chromosomes without inversions (**A**), with *In(1)sc^8^* (**B**) and with both *In(1)sc^8^* and *In(1)19EHet* inversions (**C**). Schemes of polytene chromosomes are given for the *Rif1^+^* and *Rif1^-^* backgrounds. Positions of *In(1)sc^8^* and *In(1)19EHet* breakpoints are marked by arrows on chromosomes that are not rearranged. Blocks of heterochromatin are indicated according to the classical map of metaphase chromosomes [7]. The colours of the 28 S rDNA and 1.688 satellite reflect the colours of FISH probes in Figure 1B. The regions affected by the new rearrangement are largely under-replicated in wild-type polytene chromosomes, and therefore, the *In(1)19Ehet* inversion could not be detected without the *Rif1^1^* background.

The *In(1)sc^8^* inversion was created in the laboratory of A. Serebrovsky by X-ray mutagenesis of *w^a^* flies [21,22,25,26,27]. The *In(1)sc^8^*, *sc^8^ y^31d^ w^a^* stock was transferred to the Bloomington Drosophila Stock Center (BDSC) from the California Institute of Technology (Caltech) in 1986 and assigned the stock number 798. In 2012, the stock was split into two independent sublines (hereinafter, #798 main copy and backup copy). In 2020, we obtained flies of the #798 main copy stock from the BDSC and founded a laboratory subline (hereinafter, #798 2020 subline). Then, we performed genetic crosses to combine the X chromosome from the #798 2020 subline with the second chromosome carrying the *Rif1^1^* mutation to obtain a new stock with the genotype *In(1)sc^8^*, *sc^8^ y^31d^ w ^a^*; *Rif1^1^*. The *Rif1^1^* mutation completely suppresses underreplication in the pericentromeric and intercalary heterochromatin [37,38]. Therefore, the *Rif1^1^* mutant background allows us to observe all *D. melanogaster* heterochromatic regions on polytene chromosome preparations. Morphological analysis of polytene chromosome squashes obtained from the new *In(1)sc^8^*, *sc^8^ y^31d^ w ^a^*; *Rif1^1^* subline demonstrated the presence of an additional rearrangement on the X chromosome. In phase-contrast images after aceto-orcein staining, part of the X chromosome—including the proximal end of region 19, region 20, and a significant part of the X heterochromatin—appeared to be inverted (Figure 1A) compared to the known *In(1)sc^8^* rearrangement, which has been previously characterized by polytene chromosome and genomic sequence analyses. After analyzing about fifty X chromosomes on ten polytene chromosome squashes, we were able to localize one of the new inversion breakpoints to the proximal end of the intercalary heterochromatin band 19E1–4 (Figure 1A). The second breakpoint is positioned deep in heterochromatin, very close to the heterochromatic breakpoint of the progenitor *In(1)sc^8^* inversion. We named the new inversion *In(1)19Ehet* and the complex chromosomal rearrangement (both inversions) *In(1)sc^8^ + 19EHet.* Schematic diagrams are shown in Figure 2. To better understand the exact breakpoint position in heterochromatin, we performed FISH with probes for rDNA and a heterochromatic variant of the 1.688 satellite and confirmed that the 11 Mb X chromosome 1.688 satellite block is broken into three blocks by the *In(1)sc^8^ + 19EHet* double inversion (Figure 1B). The largest block is transferred to the 19E region and lies between 19E and the nucleolus. Another block remains near the centromere of the X chromosome. A small block remains near the *In(1)sc^8^* breakpoint at the telomeric end of the chromosome (Figure 1B).

To determine the origin of *In(1)19EHet*, we checked for the presence of the inversion in both the main and backup copies of the #798 line at the BDSC. Without the *Rif1* mutant background, it is impossible to accurately confirm the presence of a rearrangement in heterochromatin due to its underreplication (see Figure S1A as an example of the morphology of the X chromosome for the #798 main copy subline without the *Rif1^1^* mutation). Therefore, we crossed both sublines with the line carrying the *Rif1^1^* mutation. On polytene chromosome squashes of the heterozygous offspring resulting from crosses, we clearly saw that only the #798 main copy subline had the double inversion, while the #798 backup copy subline had only the *In(1)sc^8^* inversion (Figure S1B,C). Thus, we conclude that the inversion event occurred in the #798 main copy subline after 2012 when the #798 stock was split.

### 3.2. Detection of Inversion Breakpoints Using Nanopore Sequencing

**Figure 3:**
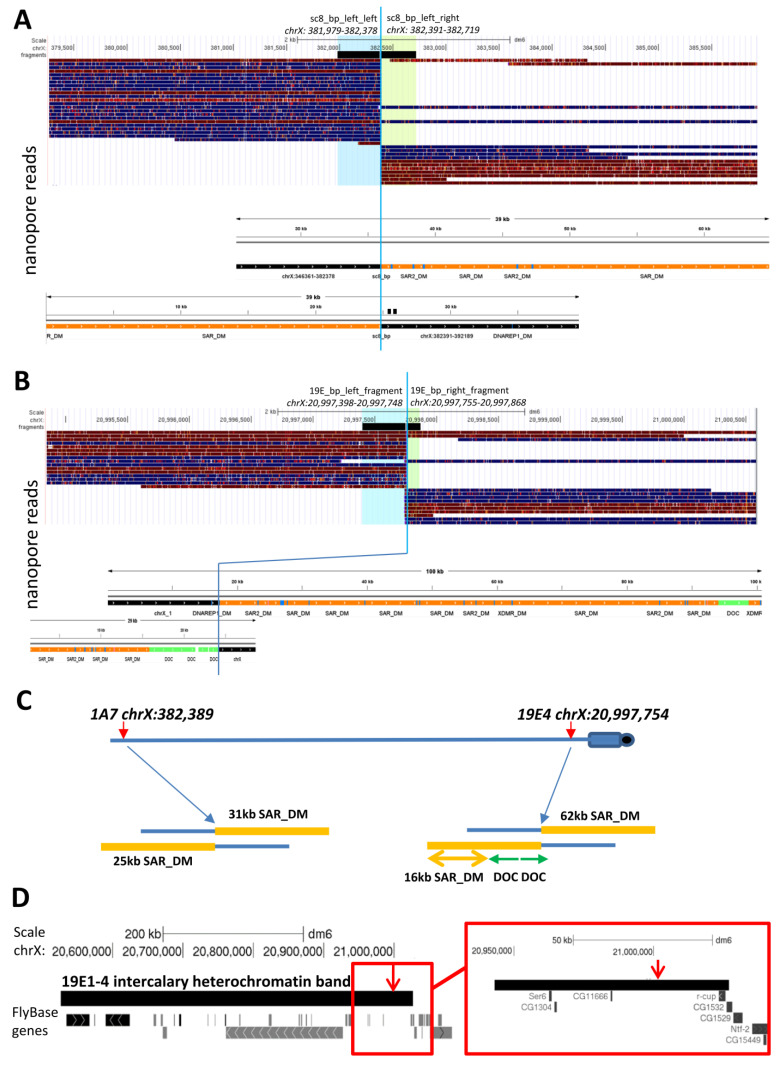
Elicitation of inversion breakpoints using nanopore sequencing. (**A**) Genome Browser view of the distal *In(1)sc^8^* breakpoint (blue line, *chrX:382,384–382,389*). Positions of two regions (*chrX: 381,979–382,378* and *chrX: 382,391–382,719*) used for extraction of nanopore reads are shown at the top. Soft-clipped aligned reads, overlapping the breakpoint, are shown below. The position and feature maps of the two longest reads overlapping the *In(1)sc^8^* breakpoint are shown below the alignment. In both cases, long (>20 kb) stretches of 1.688 satellite (designated as SAR_DM and SAR2_DM according to RepeatMasker) were detected beyond the breakpoint position. Parts of the chimeric reads corresponding to euchromatin sequence immediately near the breakpoint are marked in black. Other colors demark different types of repeats in the fused heterochromatin. (**B**) *In(1)19EHet* breakpoint in *chrX:20,997,754*. Overall arrangement of the image is the same as for A. (**C**) Schematic representation of repetitive sequences flanking *In(1)sc^8^* and *In(1)19EHet* breakpoints. Transposable elements are designated according to RepeatMasker. (**D**) The *chrX:20,997,754* breakpoint position relative to the intercalary heterochromatin polytene chromosome double band 19E1–4 and nearest gene positions.

To investigate the *In(1)sc^8^ + 19EHet* chromosome at the molecular level, we performed nanopore sequencing of the corresponding genome. We obtained a dataset with about 20-fold coverage of the *D. melanogaster* genome, with half of the reads being longer than 40 kb. *In(1)sc^8^ + 19EHet* is a derivative of *In(1)sc^8^*, and therefore, it should contain the original breakpoints of *In(1)sc^8^*, which were mapped previously [28]. According to Miller et al., the distal breakpoint is located at chrX:382,384–382,389 (*dm6*), while the proximal breakpoint occurred somewhere in pericentric heterochromatin in the 1.688 satellite block. Indeed, we found a gap in coverage by reads in the predicted position (*chrX:382*,*384–382*,*389* region) (Figure 3A). Thus, we confirmed that *In(1)19EHet* arose on a chromosome carrying *In(1)sc^8^*. We also found chimeric reads overlapping the *In(1)sc^8^* breakpoint. The two longest reads, encompassing ~30 kb of heterochromatin sequences, were selected for analysis (Figure 3A). These reads contain 1.688 satellite sequences (designated as SAR_DM or SAR2_DM in Figure 3A) juxtaposed to euchromatic regions (Figure 2A). Our analysis showed that the heterochromatin beyond the *In(1)sc^8^* breakpoint is represented by a long (>50 kb) region of a 1.688 satellite, but we identified no sequence features allowing us to locate the heterochromatic breakpoint on the genome map.

Since our cytological analysis placed one of the *In(1)19EHet* breakpoints in the 19E1–4 polytene chromosome region (Figure 1A), we analyzed nanopore reads in this region to identify the new breakpoint. As for *In(1)sc^8^*, we inspected the sequence alignments and identified an interruption in coverage at position *chrX:20,997,754* (Figure 3B). Two reads overlapping the expected breakpoint were selected and analyzed by RepeatMasker (Figure 3B) to place the *In(1)19EHet* breakpoint at position *chrX:20,997,754*, about 30 kb from the border of the 19E1–4 intercalary heterochromatin region (according to [47]) in a long (~30 kb) intergenic interval between the genes *CG11666* and *r-cup* (Figure 3D). The heterochromatin breakpoint of *In(1)19EHet* lies in a region composed of a 1.688 satellite with occasional insertions of DOC transposable elements.

**Figure 4:**
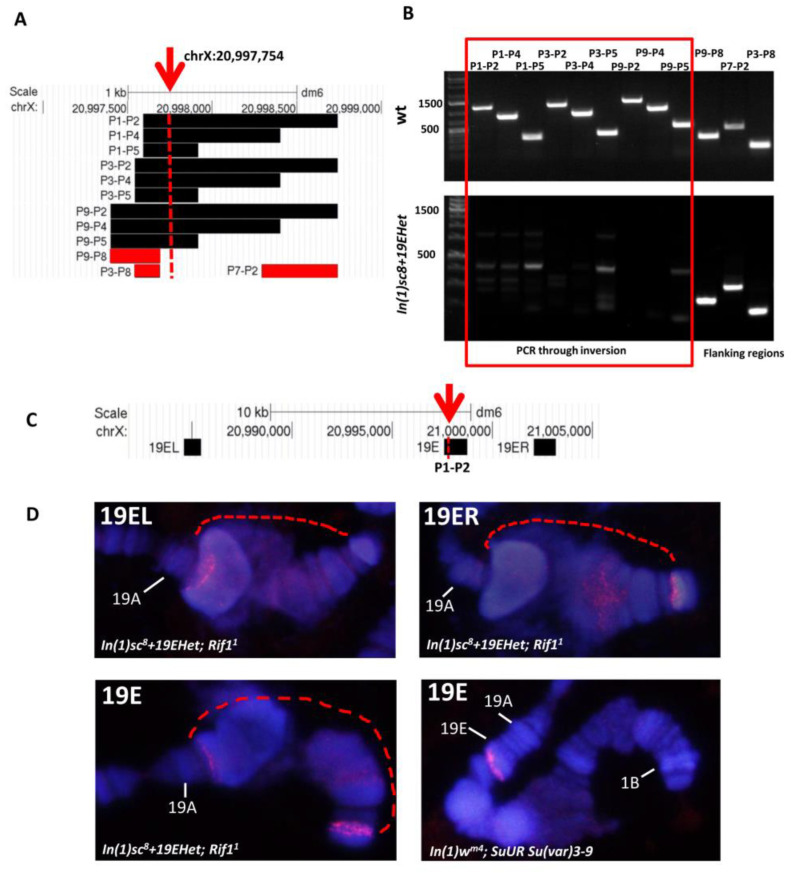
Verification of the *In(1)19EHet* euchromatin breakpoint position at *chrX:20,997,754* predicted by nanopore sequencing. (**A**) Position of PCR products obtained with various combinations of P1–P9 primers on the genomic map. The arrow shows the position of the breakpoint at *chrX:20,997,754*; black and red boxes indicate PCR products that overlap or do not overlap the inversion breakpoint, respectively. (**B**) Results of PCR with primers P1–P9. Genomic DNA samples from wild-type (upper panel) and *In(1)sc^8^ + 19EHet* (#798 2020 stock) (bottom panel) flies were used as templates. PCR results for primer combinations overlapping the breakpoint are indicated by the red box. (**C**) Position of DNA FISH probes 19EL, 19E and 19ER on the genomic map. The arrow shows the *chrX:20,997,754* breakpoint. (**D**) FISH with *In(1)sc^8^ + 19EHet; Rif1^1^* and control (*In(1)w^m4h^; SuUR Su(var)3-9*) polytene chromosomes. The dashed line shows the inverted area. The 19EL and 19ER probes hybridize to chromosomal regions separated by the inversion event. 19E probe, broken by inversion, hybridizes on both sides of the break with the brightness of the signals corresponding to the ratio of fragment sizes. In the control line, a single probe signal 19E marks the proximal edge of the 19E region. *SuUR Su(var)3-9* background in control line shows underreplication similar to *Rif1* in 19E1–4.

To verify the *chrX:20*,*997*,*754* breakpoint predicted by nanopore sequencing, we performed PCR on DNA isolated from *In(1)sc^8^ + 19EHet* and wild-type flies using different combinations of P1–P9 primers (see materials and methods) (Figure 4A,B). PCR on genomic DNA from wild-type flies produced amplicons of the expected lengths with all primers used (Figure 4B, upper panel). PCR on DNA from *In(1)19Ehet* flies produced amplicons with primers from regions upstream (P9–P8, P3–P8 combinations) and downstream (P7–P2) of the breakpoint. No specific PCR products were observed during amplification with primers flanking the breakpoint (Figure 4B, bottom panel). We also confirmed our mapping by DNA FISH with three probes, one of which overlapped the supposed breakpoint (19E probe obtained with P1 and P2 primers), and with the other two lying to the right and left of the breakpoint within 10 kb (Figure 4C,D). The *In(1)w4* inverted chromosome was used for a control to demonstrate the absence of a signal in the 1AB region. The *SuUR Su(var)3-9* background was used to suppress under-replication in the 19E intercalary heterochromatin region.

According to our cytological analysis, the additional inversion was present in the #798 main copy stock in BDSC but absent in the #798 backup copy stock. In order to obtain pure stocks carrying only the *In(1)sc^8^ + 19EHet* double inversion chromosome, we made ~30 matings of single females to single males from the #798 main copy. PCR amplifications from a sampling of these lines confirmed that the double-inverted X chromosome had completely replaced the single-inverted X chromosome in the #798 main copy stock (Figure S2). Two stocks were thus distributed by the BDSC: the *In(1)sc^8^*, *y^31d^ sc^8^ w^a^* stock (originally the backup copy) retained stock number 798, while *In(1)sc^8^*, *In(1)19EHet*, *y^31d^ sc^8^ w^a^* (one of the 798 main copy lines validated by PCR) was assigned stock number 94727.

### 3.3. Morphology of the Nucleolar Organizer in In(1)sc^8^ + 19EHet; Rif1^1^ Polytene Chromosomes

**Figure 5:**
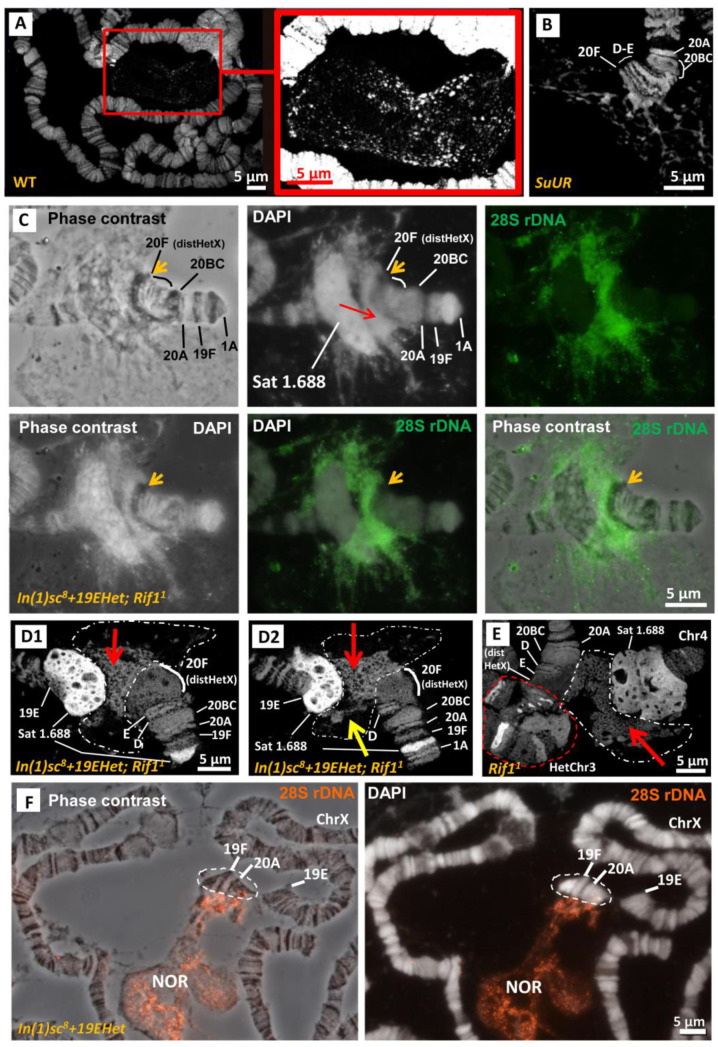
Comparison of the nucleolus morphology in wild-type, *SuUR* and *In(1)sc^8^ + 19EHet; Rif1^1^* polytene chromosomes. (**A**) DAPI staining with subsequent 3D-SIM microscopy of wild-type polytene chromosomes. The nucleolus is not closely associated with the proximal end of the X chromosome but is connected to the chromocenter and other chromosome regions. The inset shows the nucleolus clearer via overexposure. (**B**) DAPI staining and 3D-SIM of *SuUR* mutant polytene chromosomes. (**C**) FISH with 28S rDNA probe (green) in *In(1)sc^8^ + 19EHet*; *Rif1^1^* polytene chromosomes. Chromosome morphology is shown by phase-contrast imaging and DAPI staining. The red arrow indicates the intrachromosomal rDNA FISH signal. The orange arrow indicates the distal heterochromatin (distHetX) in band 20F poorly stained by DAPI. (**D1**) DAPI staining and 3D-SIM analysis of *In(1)sc^8^ + 19EHet*; *Rif1^1^* chromosomes. The intrachromosomal part of the NO is represented by a spongy structure (indicated by red arrow) located between the block of 1.688 satellite, which is strongly stained by DAPI, and distHetX, which is poorly stained by DAPI. The boundaries of the nucleolus, which consists of the spongy structure and a cloud of DAPI-stained specks, are shown by the white dotted line. The yellow arrow indicates filaments connecting the spongy structure with the cloud. (**D2**) Another 3D-SIM slice. (**E**) DAPI staining and 3D-SIM analysis of uninverted chromosomes in a *Rif1^1^* mutant showing the morphology of X chromosome heterochromatin similar to (D). Contacts with heterochromatin of other chromosomes (red dotted line) complicate the analysis. (**F**) FISH with 28S rDNA probe (green) in polytene chromosomes of *In(1)sc^8^ + 19EHet* without *Rif1^1^* mutation. Chromosome morphology is shown by phase-contrast imaging and DAPI staining. The polytenized part of the inverted by 19EHet X chromosome fragment is indicated by a dotted line.

In preparations of wild-type polytene chromosomes, the nucleolus usually lies outside the chromosomes (Figure 5A). Mutations that prevent the underreplication of heterochromatin may allow us to visualize a part of the nucleolus organizer region (NOR) that is normally hidden as well as adjacent chromosome regions. In preparations of *SuUR* mutant chromosomes, which undergo additional polytenization of some pericentromeric sequences [5,48], the nucleolus is connected with distal heterochromatin by DAPI-stained strands (Figure 5B), but it still appears as a “cloud” of granules lying outside of the chromosome. In lines with *Rif1* mutations, the chromosomes are completely polytenized, and the NOR does not separate from the X chromosome [38], but visualization of the nucleolus is hampered by the fact that the NOR and neighboring sequences are usually combined into a single chromocenter together with heterochromatin regions belonging to other chromosomes (not shown). Because the NOR and most pericentromeric heterochromatin are relocated to a position near the X tip by the *In(1)sc^8^* and *19EHet* inversions, these regions are often positioned away from the chromocenter in preparations made from *Rif1^1^* mutants (Figure S3). Thus, the combination of the *In(1)sc^8^ + 19EHet* chromosome and the *Rif11* mutation can be used as a suitable model system for standalone imaging of fully replicated NOR and adjacent heterochromatin regions.

Figure 5C shows the result of 28S rDNA FISH on *In(1)sc^8^ + 19EHet*; *Rif1^1^* polytene chromosomes. The FISH signal was detected between the large block of chromatin stained strongly with DAPI corresponding to the 1.688 satellite and a distal heterochromatin block, which is very compact on the phase-contrast image and not stained brightly by DAPI (orange arrows in Figure 5C). This block of distal heterochromatin is usually designated as 20F and corresponds to the region on the genomic map lying proximal to the *su(f)* gene mapped in 20E [2,5]. The rDNA FISH signal is much wider than the DAPI-stained chromosome and also forms a cloud outside of the chromosome territory (Figure 5C), where it strongly coincides with the DAPI-stained network of filaments and granules (Figure S4). In this particular aspect, the structure of the nucleolar cloud of *Rif1^1^* mutants retains similarity with wild-type preparations (Figure 5A and Figure S4).

DAPI staining with 3D-SIM microscopy of *In(1)sc^8^ + 19EHet*; *Rif1^1^*(Figure 5D) and *Rif1^1^* (Figure 5E) preparations revealed that the nucleolar part of the chromosome forms an unusual spongy structure (red arrows in Figure 5D,E), which is located in the same place as the rDNA FISH signal (Figure 5C). In some 3D-SIM images, we observed filaments connecting this spongy zone of NOR with the surrounding cloud of individual DAPI dots (Figure 5D, yellow arrow). Since this spongy structure has never been observed in wild-type chromosomes and is not found in *In(1)sc^8^ + 19EHet* line without *Rif1^+^* mutation (Figure 5F), we suggested it arises due to the loss of the underreplication of a substantial part of the rDNA cluster in *Rif1* mutants. Therefore, the spongy structure likely consists of rDNA units that are not normally polytenized. Of note, this structure is absent in *SuUR* mutants (Figure 5B), in which the level of centromeric heterochromatin polytenization increases less than in the *Rif1^1^* mutants.

### 3.4. Identification of the Transcriptionally Active Part of the Nucleolus in In(1)sc^8^ + 19EHet; Rif1^1^ Polytene Chromosomes

**Figure 6:**
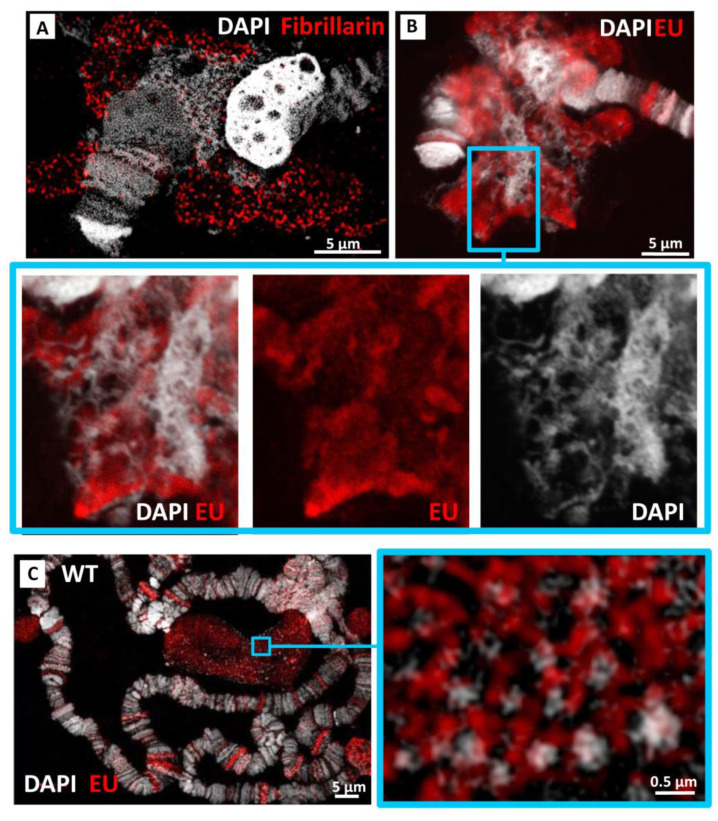
Fibrillarin and nascent transcripts are localized predominantly in the extrachromosomal part of nucleoli in *In(1)sc^8^ + 19EHet*; *Rif1^1^* polytene chromosome preparations. (**A**) Fibrillarin immunostaining in *In(1)sc^8^ + 19EHet*; *Rif1^1^* chromosome. (**B**) *In(1)sc^8^ + 19EHet*; *Rif1^1^* chromosome after a 15-min EU pulse. (**C**) EU incorporation assay in the nucleolus of wild-type chromosome preparations. A and C images represent single 3D-SIM slices. B image represents a confocal slice.

It is well known that only a part of *D. melanogaster* rDNA repeats is transcriptionally active, while other rDNA units, including those containing insertions of retroelements, are in a silent state [49,50,51,52]. In preparations of the wild-type salivary gland nucleolus, individual DAPI-stained specks have been shown to correspond to silent rDNA units enriched with repressive chromatin marks, whereas transcriptionally active units were mixed with them and poorly stained by DAPI [53]. To reveal functional areas of the unusual nucleolus in *Rif1* mutants, we visualized several nucleolar markers. Immunostaining for fibrillarin, an rRNA 2′-O-methyltransferase involved in post-transcriptional rRNA processing, showed its predominant localization in the extrachromosomal zone, mostly in the outer part of the cloud (Figure 6A), which may correspond to the dense fibrillar component of the nucleolus. Nucleolar transcripts detected by 5-Ethynyl Uridine (EU) incorporation also produced more intense signals in the cloud than in the intrachromosomal spongy structure (Figure 6B) and did not coincide with DAPI-dense regions. However, it should be noted that within 20 min of EU incorporation, nascent transcripts may have time to transfer to the processing zone. In the chromosomes of wild-type larvae, EU signals also mostly do not coincide with dense DAPI granules in the nucleolus (Figure 6C).

**Figure 7:**
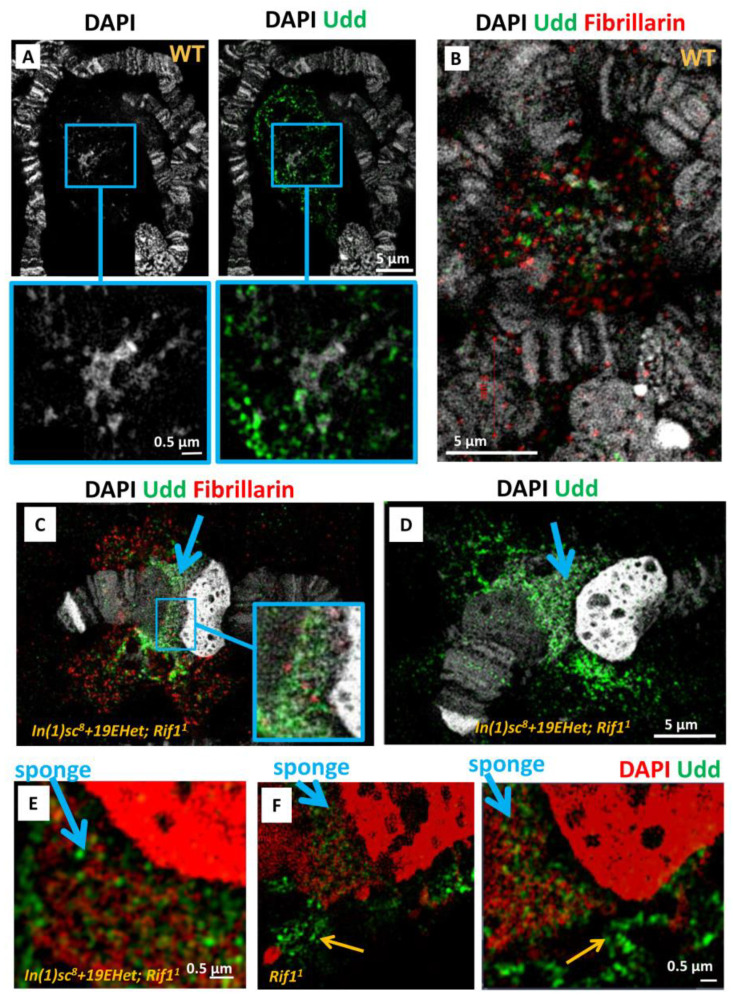
Udd is present both in the spongy structure and in the extrachromosomal cloud of the NOR. (**A**) Wild-type polytene chromosomes stained with DAPI and Udd antibodies. (**B**) Udd, fibrillarin and DAPI localization in wild-type polytene chromosomes. (**C**) *In(1)sc^8^ + 19EHet; Rif1^1^* chromosomes immunostained for Udd and fibrillarin. (**D**) Same as (**B**) without fibrillarin. (**E**) Close-up view from (**D**) showing Udd dots in the spongy area (blue arrows) and in the cloud (yellow arrows). (**F**) *Rif1^1^* chromosomes immunostained for Udd (all designations correspond to (**E**)). All images represent slices from 3D-SIM image stacks.

To identify which part of the nucleolus in the polytene chromosome preparations corresponds to the zone of active DNA transcription, we performed immunostaining to identify Underdeveloped (Udd), a component of the rDNA transcription initiation complex SL1-like [46]. Udd was shown to be associated with the IGS regions and rDNA promoters of transcriptionally active rDNA units [52]. In the nucleoli of wild-type salivary glands, Udd aggregates mostly did not co-localize with the DAPI-stained specks (Figure 7A). Additionally, Udd signals did not coincide with fibrillarin-positive dots (Figure 7B), indicating the existence of multiple separate zones responsible for transcription or rRNA processing. In the wild type, these compartments seem to be intermingled (Figure 7B). In contrast, in *In(1)sc^8^ + 19EHet; Rif1^1^* nucleoli, Udd-stained specks appeared to be clustered separately from fibrillarin-stained specks (Figure 7C). Udd is localized in the form of separate dots both in the intrachromosomal spongy zone and in the cloud (Figure 7D). In the cloud, it is often amassed into larger spots surrounded by fibrillarin (Figure 7C). At higher magnification, it is noticeable that Udd mostly does not co-localize with condensed DAPI specks in the cloud (Figure 7E,F, orange arrows). Within the spongy structure, Udd resides predominantly in voids formed by the DAPI-stained network (Figure 7E,F, blue arrows). These data are in good agreement with the notion that condensed DAPI-stained specks correspond to transcriptionally silent rDNA units [53]. Note that Figure 7F shows immunostaining of the *Rif11* line without inversions, confirming that the spongy structure with Udd foci is a feature of the *Rif11* mutation and not the result of a chromosomal rearrangement.

Thus, we suggest that the spongy intrachromosomal part of the *Rif1^1^* mutant nucleolus consists of a mixture of active Udd-positive and inactive, condensed, Udd-negative rDNA repeats aggregated into a single structure as a result of complete rDNA replication. In contrast, the cloud is composed of predominantly active rDNA units looped out from the chromosomal NOR. These units are surrounded by an rRNA processing apparatus, an analogue of the dense fibrillar component of the nucleolus, which is detected by fibrillarin staining.

### 3.5. The In(1)sc^8^ + 19EHet; Rif1^1^ Genotype Allows Visualization of the Y Chromosome Nucleolus

**Figure 8:**
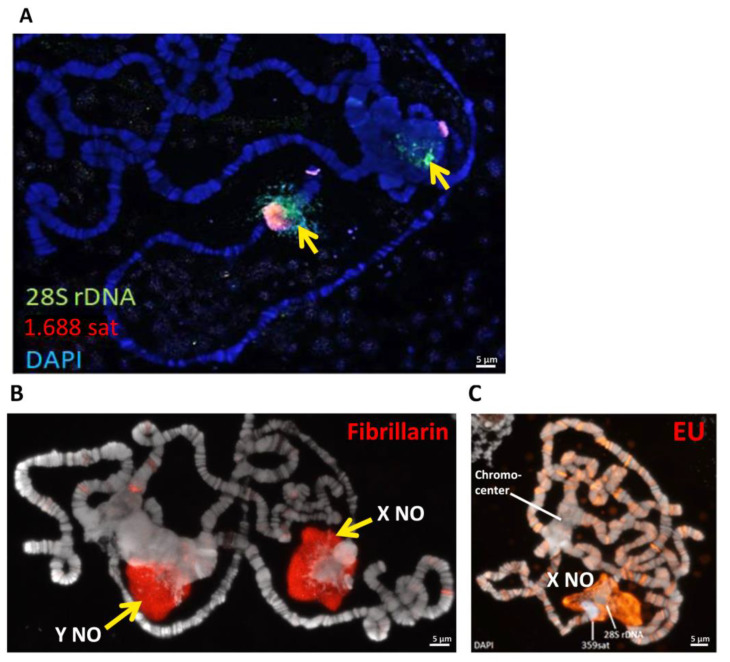
Detection of X- and Y-chromosome nucleoli in polytene chromosomes of *In(1)sc^8^ + 19EHet*; *Rif1^1^* male larvae. (**A**) FISH of 28S rDNA (green) and 1.688 satellite (red) probes. A large block of 1.688 satellite marks X-chromosome heterochromatin removed from the chromocenter by *In(1)sc^8^ + 19EHet*. Two rDNA clusters can be seen (arrows). (**B**) An example of two active nucleoli visualized by fibrillarin immunostaining. (**C**) An example of an active X chromosome nucleolus and an inactive Y chromosome nucleolus visualized by EU pulse incorporation.

In males of *D. melanogaster*, the nucleolar organizer is present on both the X and Y chromosomes. In some cases, both nucleoli are active, while in others, an active nucleolus is formed only on one of the chromosomes—a phenomenon known as nucleolar dominance [19,20,21,54]. In polytene chromosomes of salivary glands, the Y chromosome is usually not polytenized and is not visible. However, genotypes with inversions that transfer Xh from the centromere made it possible to see the nucleolus derived from the Y chromosome in the chromocenter [4]. FISH with a 28S rDNA probe on the polytene chromosomes of *In(1)sc^8^ + 19EHet/Y*; *Rif1^1^* males revealed two loci: the X-chromosome nucleolus moved away from the chromocenter and a zone in the chromocenter (Figure 8A). This chromocenter zone likely corresponds to the Y chromosome rDNA cluster. An analysis of 10 preparations of *In(1)sc^8^ + 19EHet/Y*; *Rif1^1^* larvae by fibrillarin immunostaining or EU detection showed that the X chromosome nucleolus is active in all analyzed preparations, whereas the Y chromosome nucleolus was active in half of the cases (as exemplified in Figure 8B) and inactive in the others (as shown in Figure 8C). Further analysis of *In(1)sc^8^ + 19EHet/Y*; *Rif1^1^* polytene chromosomes may help identify new factors that affect the activity of the Y-chromosome nucleolus.

## 4. Discussion

Eu-heterochromatin inversions are classic tools for investigating position effect variegation, the interaction between eu- and heterochromatin, and chromatin organization in general. In particular, many heterochromatin proteins, such as HP1 and *Su(var)3-9* histone methyltransferase, were discovered as position-effect variegation modifiers [55,56,57]. Here, we have shown that a new inversion, *In(1)19EHet*, arose spontaneously in a stock already carrying the classical *In(1)sc^8^* inversion (the BDSC #798 line [28]) after the split of two sublines in 2012. Since then, the double inversion chromosome has completely replaced the *In(1)sc^8^* single chromosome in the stock population. The new *In(1)19EHet* rearrangement transferred most of the X chromosome heterochromatin, including the rDNA cluster, to a new euchromatic environment in the 19E region (Figure 2). The *In(1)19EHet* inversion has a number of properties, which, in particular, make it possible to conveniently study the structure of the nucleolus, which appears to be remote from the bulk of heterochromatin belonging to other chromosomes. Although we did not directly compare the localization of the nucleoli between *In(1)sc^8^* and *In(1)sc^8^ + 19EHet* lines, we suggest that the *In(1)19EHet* rearrangement may be more convenient for nucleolus analysis because the transfer of NOR from the telomeric end of the chromosome more than 3 Mb deep into euchromatin can further reduce the frequency of the association of the nucleolus with the heterochromatin of other chromosomes.

The detection of the *In(1)19EHet* inversion was made possible by the unique effect of the *Rif1^1^* mutation, which leads to the complete replication of heterochromatin sequences in polytene chromosomes. Our results show that *Rif1* mutations are a useful tool for *Drosophila* heterochromatin studies. Even in a heterozygote, *Rif1^1^* allows visualization of many more heterochromatin regions than can be seen in wild-type larvae. This distinguishes it from *SuUR* mutations and *SuUR* mutations in combination with *Su(var)3-9* mutation, which both have significant effects on heterochromatin underreplication only in homozygotes [48,58,59].

The combination of the new *In(1)19EHet* inversion and the *Rif1^1^* mutation in the homozygous state allows us to visualize fully replicated NORs and adjacent X heterochromatin regions apart from other chromosomes. In wild-type polytene chromosome preparations, the nucleolus is a cloud-like structure that is found separately from the chromosomal material, to which it is connected by thin fibers. In *Rif1* mutants, an intrachromosomal part of the nucleolus appears in addition to the nucleolar cloud, similar in structure to a normal nucleolus. We have found that the unusual intrachromosomal part looks like a sponge by DAPI staining and spatial super-resolution SIM imaging and that it contains rDNA repeats as identified by FISH (Figure 5). A comparison of the ratio of rDNA to genomic DNA in polytene and diploid cells shows that only about 1/5 of rDNA repeats are normally replicated in the polytene chromosomes of the salivary glands [60,61]. Thus, the resulting spongy intrachromosomal structure likely represents the portion of the rDNA cluster that remains underreplicated in polytene chromosomes under normal conditions. Interestingly, we found that this chromosomal part of the NOR likely consists of a mix of transcriptionally active and inactive zones, although markers of nucleolus activity indicate less activity in the chromosomal part of the NOR than in the cloud around the chromosome (Figure 7).

Further, the use of the *In(1)19EHet*; *Rif1* model may allow us to explore by FISH the distribution of specific rDNA repeats, such as those enriched for transposon insertions. This approach can help characterize the internal organization of rDNA clusters, which is currently difficult to study by other methods, such as sequencing, due to the repetitive nature of rDNA. The possible existence of functional and structural domains within rDNA clusters has been discussed in the literature [62,63]. The *In(1)19EHet*; *Rif1* model can be used to examine the localization of chromatin proteins associated with specific intranucleolar zones and adjacent heterochromatin regions, which may reflect subfunctionalization.

*D. melanogaster* males contain NORs on two different chromosomes: X and Y. Since the X chromosome-derived nucleolus in *In(1)19EHet*; *Rif1* flies is separated from the bulk of the chromocenter, this system, as well as some other inversion-based models [4], make it possible to visualize both nucleoli separately in different parts of the nucleus. Thus, the *In(1)19EHet*; *Rif1* genotype provides additional advantages for the analysis of the Y-chromosomal nucleolus of males and the phenomenon of nucleolar dominance, whereby individual rDNA loci are entirely silenced. We have seen that this genotype exhibits variations in nucleolar dominance: the X chromosome rDNA cluster appears to be constitutively active, while the Y chromosome cluster seems to be inactive in only some males (Figure 8). Past studies have indicated that nucleolar dominance is complex. In *D. melanogaster* males, the Y-chromosome nucleolus usually dominates over the X-chromosome nucleolus [19,20,21], but significant effects have been observed for age, the composition of repetitive sequences in Xh and Y heterochromatin and mutations in genes encoding heterochromatin protein [19,21,22,64,65,66]. Nucleolar dominance may even vary from tissue to tissue. In salivary glands, in contrast to many other tissues, only modest Y dominance was shown [21,22].

Chromosomal structure is known to affect nucleolar dominance. Hilliker and Appels [4] used inverted X chromosomes to search for factors influencing nucleolar dominance and saw that small Xh deletions could shift dominance from the X to the Y chromosome. We do not know if the *In(1)19EHet* rearrangement itself leads to preferential use of the X nucleolus or if complete polytenization of the rDNA clusters by the *Rif1^1^* mutation affects the formation of X versus Y nucleoli. It is clear, however, that the *In(1)sc^8^ + 19EHet*; *Rif1^1^* system provides a new and convenient model for exploring the mechanistic bases of this mysterious phenomenon.

## Figures and Tables

**Table 1:** Description of the *D. melanogaster* lines used in this work.

Stock	Origin	Genotype	Comments	Source
***Rif1^1^***	Deletion obtained via CRISPR-based mutagenesis; frameshift mutations at amino acid position 14. No detectable *Rif1* protein. FBal0343570	*w^1118^*; *Rif1^1^*	Used as a source of the *Rif1*^1^ mutation	Kindly provided by Jared Nordman [37]
**#798 “main copy” (BDSC)**	Sub-lines created in 2012 in BDSC by splitting line #798	*In(1)sc^8^*, *In(1)19EHet*, *y^31^ sc^8^ w^a^*	Originally used by us to create a line that combines *In(1)sc^8^* and the *Rif1^1^* mutation, renamed #94727 after an additional inversion was discovered	BDSC
**#798 “backup copy” (BDSC)**		*In(1)sc^8^*, *y^31^ sc^8^ w^a^*	Subline of #798, retaining the original genotype	BDSC
**#798 2020**	Sub-lines created in 2020 based on the #798 “main copy” flies obtained from BDSC			
**#94727 (BDSC)**	Renamed sub-line #798 main copy after detection of a new spontaneous inversion *In(1)19EHet*	*In(1)sc^8^*, *In(1)19EHet*, *y^31^ sc^8^ w^a^*		BDSC
**#798 (BDSC)**	The BDSC stock with *In(1)sc^8^*, *y^31^ sc^8^ w^a^* genotype. After the discovery of *In(1)19EHet* in some flies, the line was cleared of carriers of additional inversion	*In(1)sc^8^*, *y^31^ sc^8^ w^a^*		BDSC
***In(1)sc^8^ + 19EHet*; *Rif1^1^*l**	A line obtained by introducing a second chromosome from line *Rif1*1 into line #798 “main copy”	*In(1)sc^8^*, *In(1)19EHet*, *y^31^ sc^8^ w^a^*; *Rif1^1^l*	A line created for the cytological analysis of the pericentromeric heterochromatin of the X chromosome. A new inversion *In(1)19EHet* was discovered in it for the first time	This work

## Data Availability

The datasets with the results of Nanopore sequencing of *In(1)sc^8^, In(1)19EHet* fly DNA are available from the SRA (SRR22071703, SRR22071702, SRR22071691, SRR22071690). Other files are attached in the supporting material.

## Supplementary Materials

The following supporting information can be downloaded at: https://www.mdpi.com/article/10.3390/cells11233872/s1, Figure S1: Cytological verification of the original #798 sublines from the BDSC for the presence of the additional inversion *In(1)19EHet*. (A) Without suppression of underreplication, it is difficult to unambiguously judge the presence or absence of the *In(1)19EHet* inversion in the subline #798 main copy. (B,C) The results of crossing #798 backup copy (B) or #798 main copy (C) with the *Rif1^1^* mutant. The offspring carrying both the inverted X chromosomes and *Rif1^1^*/+ mutation in heterozygous states were analyzed. Partial heterochromatin polytenization in *Rif1^1^*/+ heterozygotes allowed us to conclude that only line #798 main copy carries the double inversion, while line #798 backup copy carries only the original *In(1)sc^8^* inversion. Figure S2: Examples of PCR amplifications from a sampling of lines started with single-pair matings from the 798 main copy vials. Control genotypes include flies from the 798 backup copy and Oregon-R (OR) flies. P1–P2 primers were used for PCR through the inversion breakpoint. Primers for the *white* gene were used to verify that the DNA samples could be used in PCR amplifications successfully. Figure S3: Localization of the nucleolus within nuclei of *Rif1^1^* mutants carrying wild-type (A) or *In(1)sc^8^ + 19EHet* (B) chromosomes in slightly squashed preparations of polytene chromosomes. Individual optical sections obtained by DAPI staining with subsequent 3D-SIM microscopy are shown. The nucleolus is marked by fibrillarin immunostaining. Figure S4: FISH with 28S rDNA probe (red) in *Rif1^1^* mutant and wild-type polytene chromosomes. The DAPI channel is overexposed to detect weakly stained structures. In both *Rif1^1^* and wild-type nucleoli, FISH signal strongly coincides with DAPI staining.
