# Supplementary material for: A Spontaneous Inversion of the X Chromosome Heterochromatin Provides a Tool for Studying the Structure and Activity of the Nucleolus in Drosophila melanogaster
Source: Cells. 2022 Dec 1;11(23):3872. doi: 10.3390/cells11233872 (PMC9736023; doi:10.3390/cells11233872)
Supplement: Supplementary file 1 [file cells-11-03872-s001.zip › cells-2055656-supplementary.pdf]

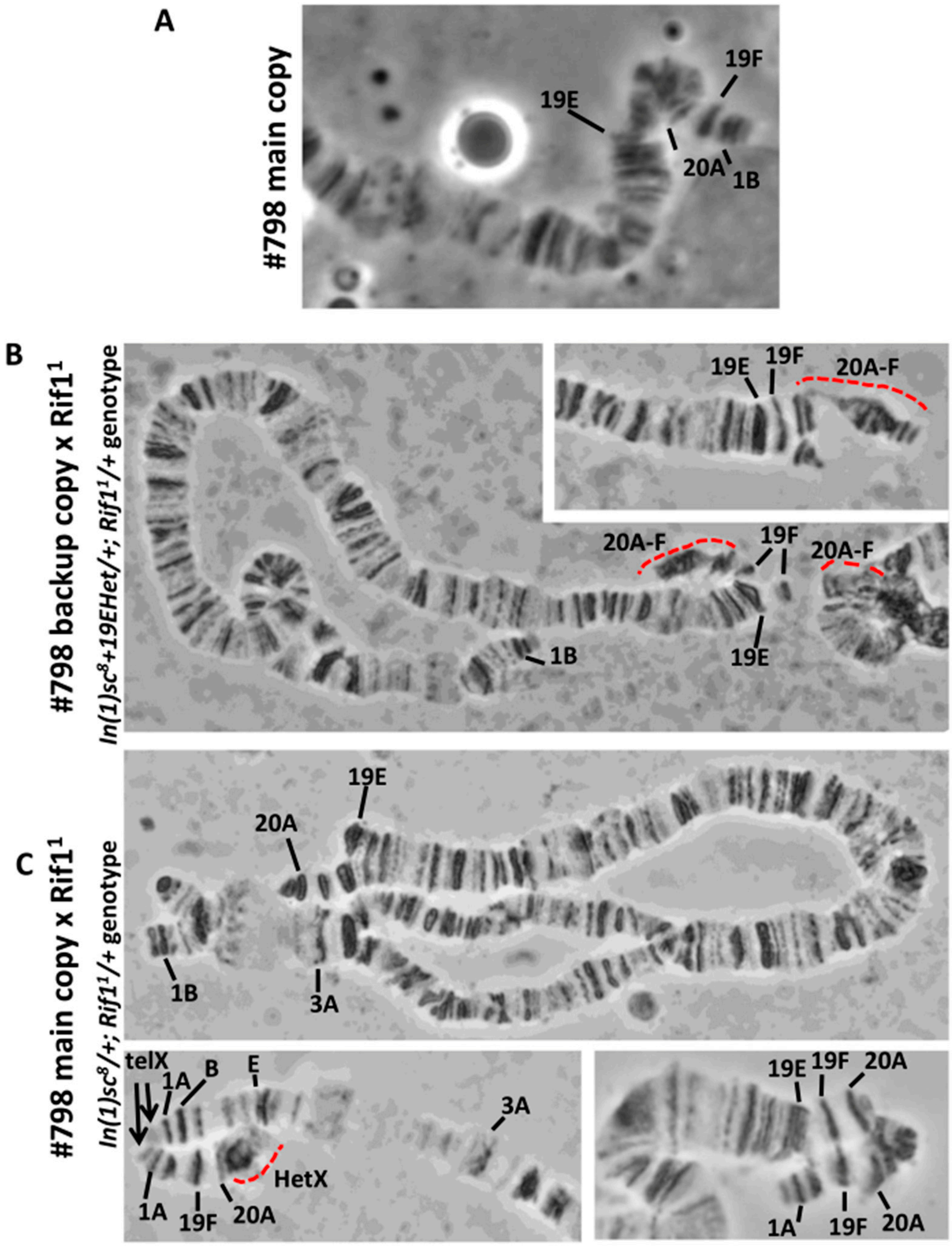

Figure S1. Cytological verification of the original #798 sublines from the BDSC for the presence of the additional inversion *In(1)19EHet*. (**A**) Without suppression of underreplication, it is difficult to unambiguously judge the presence or absence of the *In(1)19EHet* inversion in the subline #798 main copy. (B,C) The results of crossing #798 backup copy (**B**) or #798 main copy (**C**) with the *$Rif1^1$* mutant. The offspring carrying both the inverted X chromosomes and *$Rif1^1$*/+ mutation in heterozygous states were analyzed. Partial heterochromatin polytenization in *$Rif1^1$*/+ heterozygotes allowed us to conclude that only line #798 main copy carries the double inversion, while line #798 backup copy carries only the original *$In(1)sc^8$* inversion

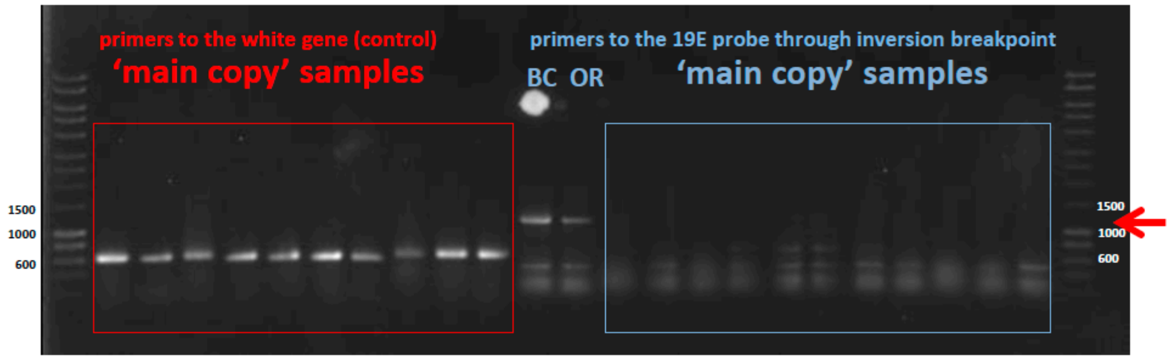

Figure S2. Examples of PCR amplifications from a sampling of lines started with single-pair matings from the 798 main copy vials. Control genotypes include flies from the 798 backup copy and Oregon-R (OR) flies. P1–P2 primers were used for PCR through the inversion breakpoint. Primers for the *white* gene were used to verify that the DNA samples could be used in PCR amplifications successfully.

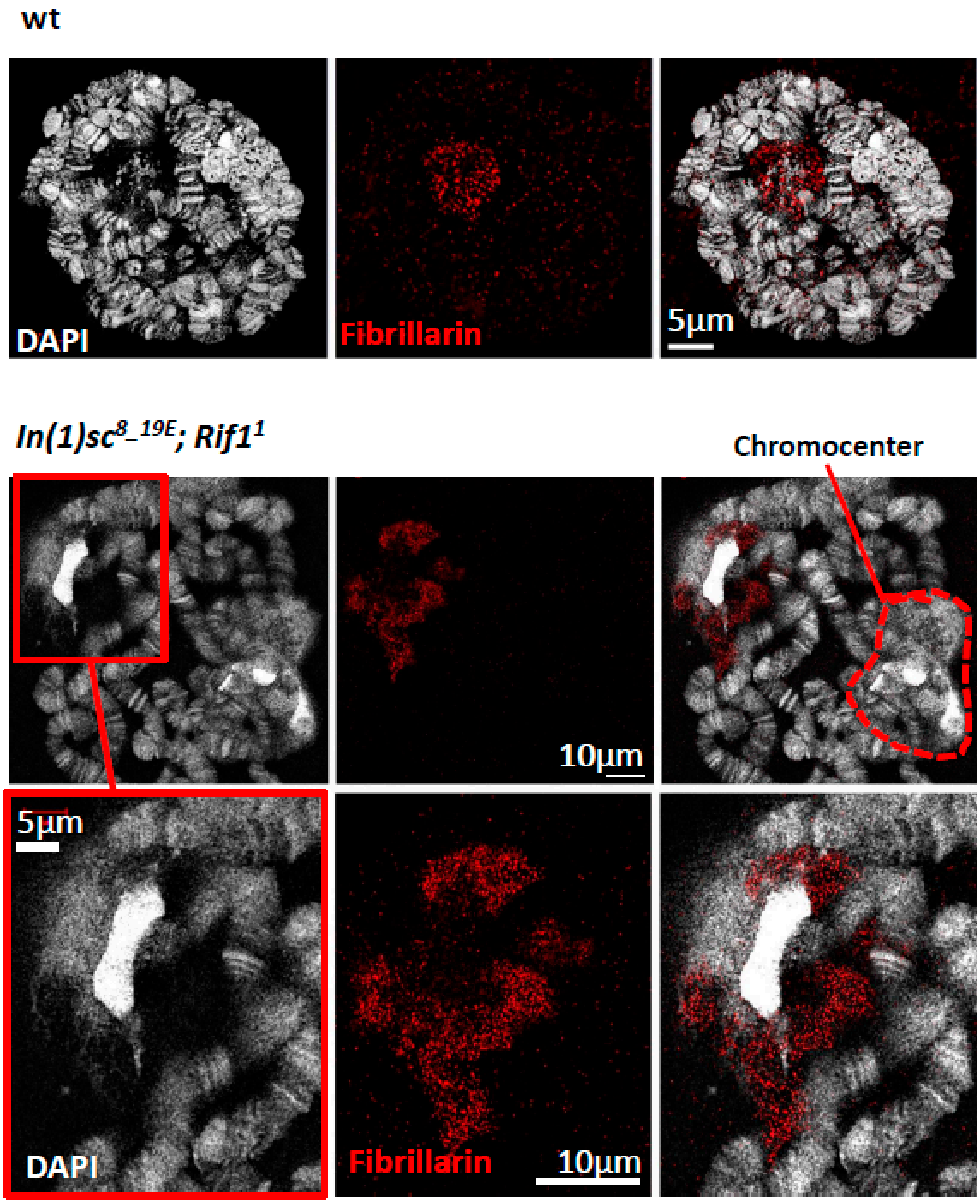

Figure S3. Localization of the nucleolus within nuclei of *Rif1*[1] mutants carrying wild-type (**A**) or *In(1)sc*[8] + *19EHet* (**B**) chromosomes in slightly squashed preparations of polytene chromosomes. Individual optical sections obtained by DAPI staining with subsequent 3D-SIM microscopy are shown. The nucleolus is marked by fibrillarin immunostaining.

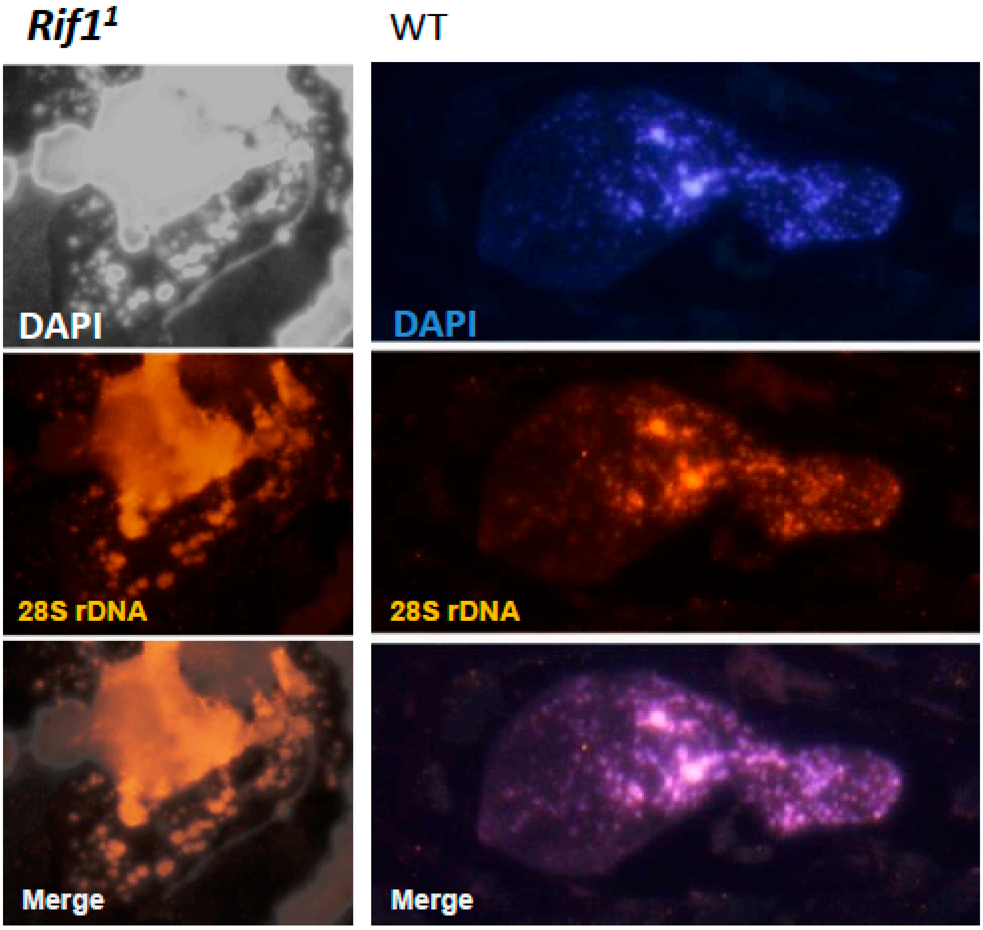

Figure S4. FISH with 28S rDNA probe (red) in *Rif1*^{1} mutant and wild-type polytene chromosomes. The DAPI channel is overexposed to detect weakly stained structures. In both *Rif1*^{1} and wild-type nucleoli, FISH signal strongly coincides with DAPI staining.
